# Neuron and astrocyte specific 5mC and 5hmC signatures of BDNF’s receptor, TrkB

**DOI:** 10.3389/fnmol.2024.1463437

**Published:** 2024-08-29

**Authors:** Xiaoran Wei, Jack L. Browning, Michelle L. Olsen

**Affiliations:** ^1^Biomedical and Veterinary Sciences Graduate Program, Virginia Tech, Blacksburg, VA, United States; ^2^School of Neuroscience, Virginia Tech, Blacksburg, VA, United States; ^3^Genetics, Bioinformatics and Computational Biology Graduate Program, Virginia Tech, Blacksburg, VA, United States

**Keywords:** neuron, astrocyte, TrkB, DNA methylation, 5-methylcytosine (5mC), 5-hydroxymethylcytosine (5hmC)

## Abstract

Brain derived neurotrophic factor (BDNF) is the most studied trophic factor in the central nervous system (CNS), and its role in the maturation of neurons, including synapse development and maintenance has been investigated intensely for over three decades. The primary receptor for BDNF is the tropomyosin receptor kinase B (TrkB), which is broadly expressed as two primary isoforms in the brain; the full length TrkB (TrkB.FL) receptor, expressed mainly in neurons and the truncated TrkB (TrkB.T1) receptor. We recently demonstrated that TrkB.T1 is predominately expressed in astrocytes, and appears critical for astrocyte morphological maturation. Given the critical role of BDNF/TrkB pathway in healthy brain development and mature CNS function, we aimed to identify molecular underpinnings of cell-type specific expression of each TrkB isoform. Using Nanopore sequencing which enables direct, long read sequencing of native DNA, we profiled DNA methylation patterns of the entire TrkB gene, *Ntrk2*, in both neurons and astrocytes. Here, we identified robust differences in cell-type specific isoform expression associated with significantly different methylation patterns of the *Ntrk2* gene in each cell type. Notably, astrocytes demonstrated lower 5mC methylation, and higher 5hmC across the entire gene when compared to neurons, including differentially methylated sites (DMSs) found in regions flanking the unique TrkB.T1 protein coding sequence (CDS). These data suggest DNA methylation patterns may provide instruction for isoform specific TrkB expression across unique CNS cell types.

## 1 Introduction

The tropomyosin receptor kinase B (TrkB) is the primary receptor for brain derived neurotrophic factor (BDNF), which is broadly expressed and released by neurons in developing and mature brains (Pandya et al., 2013). This signaling pathway plays a critical role in brain development processes, including, neuronal differentiation and survival, synaptogenesis, synaptic transmission, synaptic plasticity (Kowianski et al., 2018; Enkavi et al., 2024) as well as learning and memory (Minichiello, 2009; Bathina and Das, 2015). Given its central role in the healthy brain, it is not surprising that aberrant disrupted BDNF/TrkB signaling has been implicated in neuropsychiatric, neurodegenerative and neurodevelopmental disorders (Numakawa et al., 2018; Deng and Chen, 2024; Peng et al., 2009; Ferrer et al., 1999; Autry and Monteggia, 2012; Stansfield et al., 2012). Research in preclinical rodent studies as well as previous and ongoing clinical trials in human populations indicate targeting BDNF/TrkB signaling pathways holds therapeutic promise for neurodegenerative and neuropsychiatric diseases by driving synaptic plasticity, promoting neurogenesis, and ameliorating motor and cognitive deficits (Yoshii and Constantine-Paton, 2010; Boulle et al., 2012; Lu et al., 2013; Mitre et al., 2017; Ali et al., 2024; Enkavi et al., 2024; ClinicalTrials, n.d.).

Two main isoforms of TrkB exist in the central nervous system (CNS), the full-length receptor, TrkB.FL and the truncated receptor TrkB.T1. TrkB.FL and TrkB.T1 share 100% sequence homology in the extracellular and membrane spanning domains and thus are thought to have equal affinity for BDNF in both human and mouse (Sasi et al., 2017). TrkB.FL uniquely possesses a tyrosine kinase domain that autophosphorylates with BDNF binding to trigger multiple downstream signaling cascades, including changes in cell morphology, cell survival, neuronal differentiation and neurotransmitter release (Cao et al., 2020; Stoilov et al., 2002; Martin-Rodriguez et al., 2020; Boulanger and Poo, 1999; Huang and Reichardt, 2001). While TrkB.T1 lacks the canonical tyrosine kinase domain and much less is understood regarding the downstream signaling of BDNF binding to TrkB.T1. Previous studies have postulated that TrkB.T1 may behave as a dominant-negative receptor whereby it dimerizes with full-length TrkB to prevent autophosphorylation and canonical BDNF-mediated signaling (Fenner, 2012). Additionally, a limited number of studies have demonstrated that BDNF binding to TrkB.T1 may induce activation of protein kinase C (PKC) cascades activated by G protein signaling (Cheng et al., 2007), stimulation of phospholipase C-γ (PLCg) pathways resulting in calcium release and cytoskeletal changes (Rose et al., 2003) and inhibit Rho GTPase activity, (Ohira et al., 2005), thereby inducing cell morphological changes.

We recently demonstrated in the mouse cortex that TrkB.T1 is largely specific to astrocytes and over 90% of all *Ntrk2* expression in astrocytes is attributed to TrkB.T1 (Holt et al., 2019). This is in contrast to TrkB.FL, the predominant TrkB isoform expressed by neurons. Surprisingly, given the critical role of BDNF/TrkB signaling, few studies have attempted to identify molecular mechanisms leading to and regulating the CNS expression of TrkB isoforms (Tessarollo and Yanpallewar, 2022). Previous studies from post-mortem human frontal cortical tissue from both suicide completers and age-matched controls demonstrated lower levels of TrkB.T1 mRNA and protein correlated with altered methylation states of several CpG sites in the promoter region (Ernst et al., 2009) and 3′UTR region (Maussion et al., 2014). These studies focused on small, circumscribed, gene regions using bisulfite sequencing or microarray methodology followed by DNA methylation analysis. As indicated above, TrkB is a large, complex gene and it is unclear if methylation changes in these human studies were specific to the regions analyzed, or if changes were cell type specific.

DNA methylation, both 5mC and 5hmC, are powerful forms of epigenetic transcriptional regulation, the majority of which occurs on cytosines that precede a guanine nucleotide or CpG sites in the promoter region, gene body and intergenic gene regions (Moore et al., 2013). DNA methylation determines the spatiotemporal expression pattern of genes in most eukaryotic cells by altering transcription binding and activity, chromosomal structure, DNA conformation and DNA stability (Wang and Wu, 2018). Changes in the expression level of BDNF, the ligand of TrkB, have been observed to be linked to DNA methylation regulation (Alece Arantes Moreno et al., 2023; Ikegame et al., 2013; Mitchelmore and Gede, 2014). Importantly and relevant for this study, it has been reported that the inclusion or exclusion of nearly 20% of alternative exons in human genes is regulated by DNA methylation (Yearim et al., 2015; Ramanouskaya and Grinev, 2017). DNA methylation has been verified to collaborate with transcription factors (TFs) to regulate exon inclusion or exclusion. For example, CCCTC-binding factor (CTCF) binds to unmethylated DNA and promote the inclusion of weak upstream exons (Shukla et al., 2011) while methyl-CpG binding protein 2 (MeCP2) binds to methylated DNA to pause Pol II and leads to exon inclusion (Maunakea et al., 2013). Although less is understood about the role of 5hmC in gene isoform expression or alternative splicing, recent findings indicate that 5hmC peaks are present at the 5′ splice site of exon-intron boundaries, suggesting that 5hmC may regulate these processes (Wen et al., 2014; Khare et al., 2012).

Recently, we employed Nanopore sequencing to profile 5mC and 5hmC modifications in different CNS cell types in mouse cortex. Nanopore sequencing is a 3rd generation, long read sequencing approach which detects the electric current signatures of nucleic acids and modified nucleic acids as they pass through a nanopore. This approach allows for direct sequencing of native DNA and enables the identification of multiple DNA modifications in the same sample. These advantages make this approach ideal for quantitative assessment of DNA methylation patterns for large genomic regions or complex genes such as *Ntrk2*. Here we analyzed 5mC and 5hmC modification signatures of the *Ntrk2* gene in its entirety in enriched astrocyte and neuron cortical cell populations. Notably, we identified 98.74% of differentially methylated sites (DMSs) are hypermethylated in neurons and all the differentially hydroxymethylated sites (DhMSs) have higher 5hmC levels in astrocytes on the gene *Ntrk2*. Additionally, 37–38% of the DMSs/DhMSs are located around the unique CDS in TrkB.T1. Together, these data suggest DNA methylation patterns may provide instruction for isoform specific TrkB expression across unique CNS cell types.

## 2 Materials and methods

### 2.1 Nanopore sequencing analysis

The Nanopore sequencing data was downloaded from NAM-Me, which provides the position and quantitative 5mC and 5hmC levels of each CpG site within the gene *Ntrk2* (Chr13: 58805569–59133970). The detailed methodology for Nanopore sequencing is described in our previous publication (Wei et al., 2024). In brief, high molecular weight DNA was extracted and sheared to approximately 10 kb for sequencing on the PromethION platform at 20× depth. The raw signal data generated from sequencing were converted into nucleotide sequences and mapped to mm10 reference genome using Megalodon (v2.4.2). The 5mC and 5hmC modifications at CpG sites were identified based on electronic signal differences and mapping results, utilizing the model provided by Remora (v0.1.2). Only CpG sites with total coverage >9 and identified in at least two biological replicates were included in the following analysis. 5mC/5hmC sites were defined as sites exceeded 10% 5mC/5hmC levels (Varley et al., 2013; Ziller et al., 2013). DMS/DhMS and DMR/DhMR analysis were performed with the R package DSS (v2.44.0) with smoothing = T setting. Sites were classified as DMSs/DhMSs if they had 5mC/5hmC level difference greater than 10% and FDR less than 0.01 (Shi et al., 2021; Farlik et al., 2016). DMRs/DhMRs were defined as any region over 50 bp with at least 3 CpG sites, where more than half CpG sites in the region demonstrated as DMSs/DhMSs (Park and Wu, 2016; Feng et al., 2014). The TrkB.FL and TrkB.T1 exhibit differences in their first two exons, with varying starting and ending positions, while the CDS regions (CDS1 to CDS11) are identical between the isoforms. To avoid discrepancies in exon annotation between TrkB.T1 and TrkB.FL isoforms, we annotated DMSs/DhMSs to CDS (instead of exon), intron, 3′UTR and 5′UTR regions.

### 2.2 Animals

Wild-type C57BL/6 male mice were housed and bred at Virginia Polytechnic Institute and State University with all experiments approved by Virginia Polytechnic Institute and State University Animal Care and Use Committee. Mice were maintained on a reverse 12-h light/dark cycle (lights on at 10 pm, lights off at 10 am) with food and water available *ad libitum*. Tissue collection was conducted during dark phase between 10 am and 2 pm.

### 2.3 Sequential cell isolations

Astrocytes and neurons were isolated from cortices with magnetic cell isolation method following the previously published protocol (Holt and Olsen, 2016; Holt et al., 2019). Briefly, mice were anesthetized with CO_2_ on postnatal day 28 +/− 1 day (P28). Following dissection, cortices were processed with papain dissociation kit (Worthington Biochemical, #LK003153) to acquire single cell suspensions. Myelin+ microbeads (Miltenyi Biotech #130-096-733) and CD11b + beads (Miltenyi Biotech #130-093-634) were utilized to remove oligodendrocytes and microglia in the cell suspension. The remaining cells were captured and the cell suspension was split in half to isolate astrocytes or neurons as we have described (Holt and Olsen, 2016, Holt et al., 2019).

### 2.4 RNA isolation and qPCR

Isolated cells were stored in Trizol (−80°C) before RNA isolation. RNA was isolated using the Direct-zol RNA Microprep kit (Zymo Research, #R2060) according to the manufacturer’s instructions. Four nanograms RNA was reverse transcribed into cDNA using iScript^™^ Reverse Transcription Supermix (Bio-Red, #1708841). Here Taqman PCR master mix (Thermo Fisher Scientific, #4444557) and TaqMan probes for total TrkB (Thermo Fisher Scientific, #Mm00435422_m1), TrkB.FL (Thermo Fisher Scientific, # Mm01341761_m1) and TrkB.T1 (Thermo Fisher Scientific, TCAAGTTGGCGAGACATTCCA) were used for this purpose, with Gapdh (Thermo Fisher Scientific, #4352339E) serving as the housekeeping gene. The ddCt method was employed to determine the relative mRNA expression levels.

### 2.5 Statistics

All 5mC/5hmC level data are presented as the mean of three biological replicates. Graphs were generated using R language and GraphPad Prism version 10.2.1. Statistical significance was assessed using either the *t*-test or the Kolmogorov–Smirnov test, depending on the experimental design with *p*-value of less than 0.05 considered as the threshold for significance.

## 3 Results

### 3.1 Cell type specific expression of TrkB in the CNS

**Figure 1:**
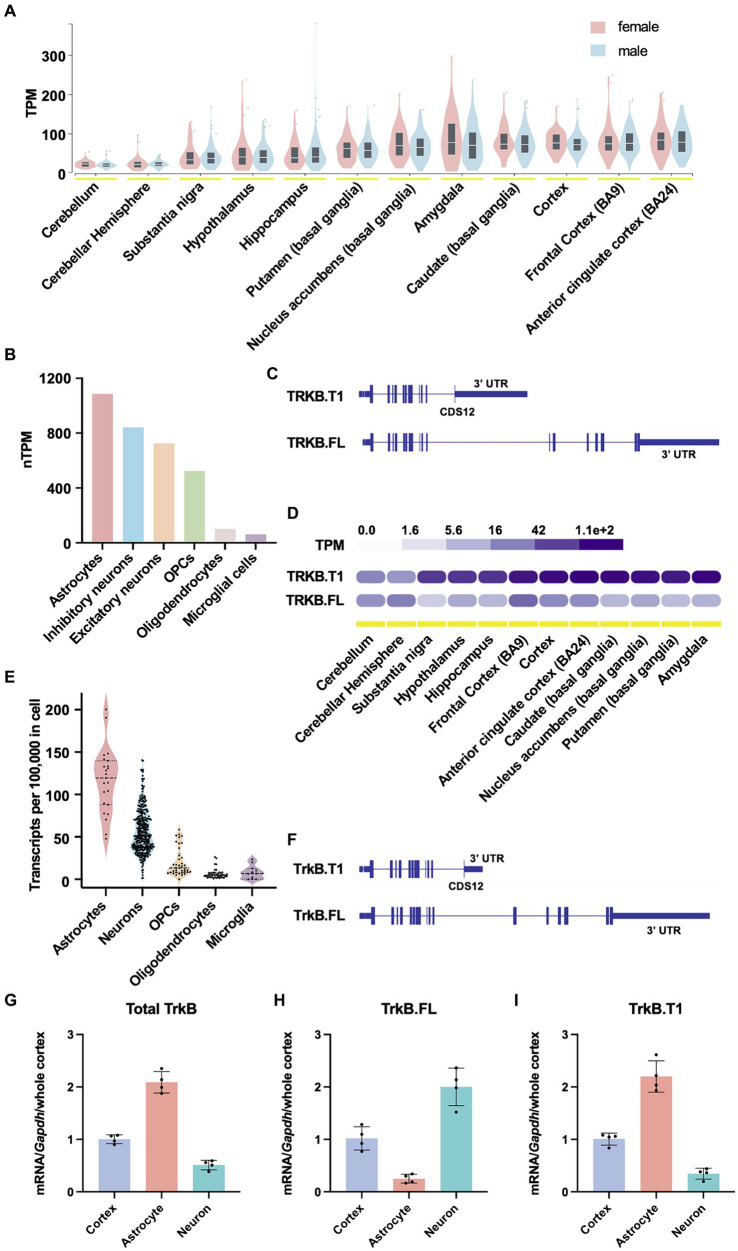
*NTRK2* expression across species. **(A)** *NTRK2* expression levels across brain regions from human bulk RNA sequencing. No sex differences in total *NTRK2* expression are observed. The data used for the analyses described in this figure were obtained from: bulk tissue gene expression for *NTRK2* in the GTEx Portal on 5/30/24. **(B)** Human *NTRK2* expression levels in unique CNS cell populations. **(C)** Human *NTRK2* gene isoform structure of the truncated (TrkB.T1) and full length (TrkB.FL) transcripts. The vertical lines represent CDS regions. **(D)** Gene expression levels of human truncated (TrkB.T1) and full length (TrkB.FL) isoforms across brain regions. The data used for the analyses described in this figure were obtained from: exon expression for *NTRK2* in the GTEx Portal on 4/5/24. **(E)** *Ntrk2* expression in unique CNS cell types obtained from mouse single cell sequencing data (Saunders et al., 2018). **(F)** Mouse *Ntrk2* isoform structure of the truncated (TrkB.T1) and full length (TrkB.FL) transcripts. **(G–I)** Quantitative PCR results obtained from in mouse cortex, isolated cortical astrocytes and isolated neurons for total TrkB **(G)**, TrkB.FL **(H)** and TrkB.T1 **(I)**. *n* = 4 animals per group.

TrkB protein is encoded by the neurotrophic receptor tyrosine kinase 2 gene (*NTRK2*), a large, complex gene. In humans, the *NTRK2* gene spans approximately 360 kb, and approximately 330 kb in mice. The protein coding sequences (CDSs) 1–11 of the *NTRK2* gene share 100% sequence homology between the full length and truncated isoforms. TrkB.FL uniquely possesses CDSs 13–19 (CDSs 13–18 in mouse), while TrkB.T1 possesses a unique CDS 12. To better understand TrkB.T1 expression across CNS brain regions, cell types and species, we first mined publicly available *NTRK2* expression data from the human protein atlas (Karlsson et al., 2021; Human Protein Atlas, n.d.) and the Genotype Tissue Expression (GTEx) portal. In humans, *NTRK2* is most highly expressed in cortex, followed by basal ganglia and amygdala regions, with no differences observed between males and females (Figure 1A, GTEx, bulk tissue expression for *NTRK2* on 5/30/24). At the single cell level, across all cell clusters, expression is highest in astrocytes, followed by inhibitory neurons, excitatory neurons, oligodendrocyte precursor cells (OPCs), oligodendrocytes and lastly microglial cells (Figure 1B, normalized HPA and GTEx transcriptomics datasets). Finally, when comparing isoform specific expression of human *NTRK2*, except for the cerebellum, which expresses overall relatively low *NTRK2*, the truncated isoform of TrkB, TrkB.T1 (477 amino acids isoform), is significantly enriched relative to TrkB.FL (838 amino acids isoform) (Figures 1C,D, GTEx portal, exon expression). Across CNS tissues, the truncated isoform of the *NTRK2* gene was expressed nearly 16-fold higher in the substantia nigra, vs. 17-fold in the amygdala and 4-10-fold in cortical brain regions when compared to the full-length isoform.

Rodent models represent the most highly studied preclinical models. Further, the vast majority of literature examining BDNF/TrkB signaling have been performed in murine models. To examine TrkB expression in mice we first evaluated single cell sequencing data from DropViz (Saunders et al., 2018). Similar to the expression pattern observed in humans, mouse *Ntrk2* exhibited the highest expression in astrocytes, with lower expression levels in neurons and other glial cells (Figure 1E). Isoform data for *Ntrk2* for mouse is not readily available, thus, to validate TrkB isoform expression in mice, we performed qPCR on enriched cortical neuron and astrocyte cell populations. To distinguish between the different TrkB isoforms, a strategic approach was employed in primer design. First, a non-specific TrkB primer spanning CDSs 9–10, common to both TrkB.FL and TrkB.T1 was designed to detect total TrkB expression. Subsequently, two additional primer sets were created for isoform-specific expression. One set spanned CDSs 11–12, which are exclusive to TrkB.T1. The other set targeted CDS 17–18, specific to TrkB.FL isoform (Figure 1F). Consistent with publicly available human data, and our previously published data (Holt et al., 2019) we observed that enriched cortical astrocytes express relatively higher levels of TrkB relative to neurons [*t*-test, *t*(6) = 14.09, *p*-value <0.0001] when normalized to whole cortical homogenates and *Gapdh* expression (Figure 1G). When evaluating isoform expression, neurons show an enrichment of TrkB.FL [*t*-test, *t*(6) = 9.533, *p*-value <0.0001] (Figure 1H), while astrocytes express predominantly express TrkB.T1 [*t*-test, *t*(6) = 11.69, *p*-value <0.0001] (Figure 1I).

### 3.2 Neurons are hypermethylated in gene *Ntrk2* compared to astrocytes

**Figure 2:**
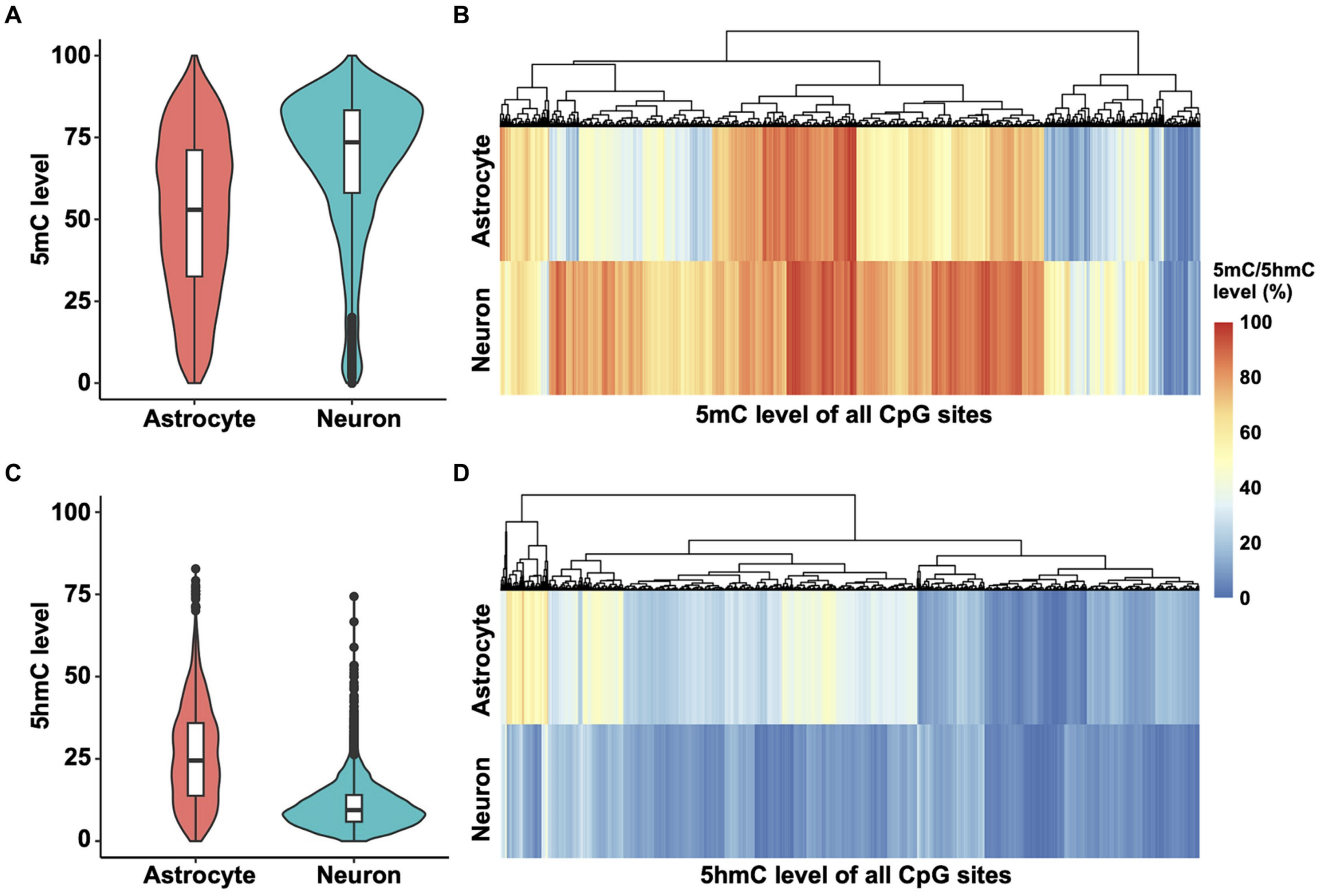
5mC and 5hmC mapping of *Ntrk2* in astrocytes and neurons. **(A,B)** Violin plots **(A)** and heatmaps **(B)** demonstrate quantification of 5mC at each CpG site of *Ntrk2* in astrocytes and neurons. **(C,D)** Violin plots **(C)** and heatmaps **(D)** demonstrate quantification of 5hmC levels on each CpG site of *Ntrk2* in astrocytes and neurons. The colors in the heatmap represent the 5mC or 5hmC levels.

To determine methylation patterns in the *Ntrk2* gene, we mined Nanopore sequencing data (Wei et al., 2024) and performed DNA 5mC and 5hmC methylation analysis. These data were obtained from enriched neuron and astrocyte cell populations from murine cortex at postnatal day 28 (P28), as presented for the qPCR results (Figure 1). We chose to assess Nanopore sequencing data as long read sequencing approaches including Nanopore and PacBio enable sequencing of large genes, or even the entire genome (Gouil and Keniry, 2019), thus, providing a feasible strategy to evaluate the Ntrk2 gene in its entirety in astrocytes and neurons. Further, this approach does not require bisulfite conversion, or chemical modification which can damage DNA, or PCR amplification, which avoids PCR amplification bias. Once sequenced, bioinformatic tools, enable base, and modified base calling, enabling simultaneous quantification of 5mC and 5hmC modifications from the same sample. For this study, we analyzed the entire *Ntrk2* gene (326 kb), and promoter region (1 kb upstream of the transcriptional start site) and 3′UTR in astrocytes and neurons. Using this approach, we observed a significant difference between the distribution of *Ntrk2* 5mC modifications in astrocyte and neuron (Kolmogorov–Smirnov test, *D* = 0.32462, *p*-value <2.2 × 10^−16^). Overall, neurons show generally higher 5mC levels compared to astrocytes (Figures 2A,B), with the median methylation level in neurons at approximately 73.5%. This value was approximately 53% in astrocytes (Figure 2A), indicating a 20% difference in DNA methylation level generally between neurons and astrocytes. Furthermore, in neurons, approximately 82% of CpG sites exhibit methylation levels exceeding 50%, while 54% of CpG sites demonstrate methylation levels over 50% in astrocytes.

We next performed a similar analysis for 5-hydroxymethylcytosine (5hmC) modifications of *Ntrk2* in astrocytes and neurons. Again, here we observed significant differences in the distribution of 5hmC modifications across *Ntrk2* in astrocytes and neurons (Kolmogorov–Smirnov test, *D* = 0.53452, *p*-value <2.2 × 10^−16^). Astrocyte *Ntrk2* demonstrated higher 5hmC levels (Figures 2C,D) when compared to neurons with an approximate 15% difference in the median 5hmC levels (astrocyte: 24.5%, neuron: 9.4%). Generally, when compared to the median 5mC level, 5hmC modifications were lower, where in neurons, over 90% of all CpG sites demonstrated 5hmC levels below 20%.

**Figure 3:**
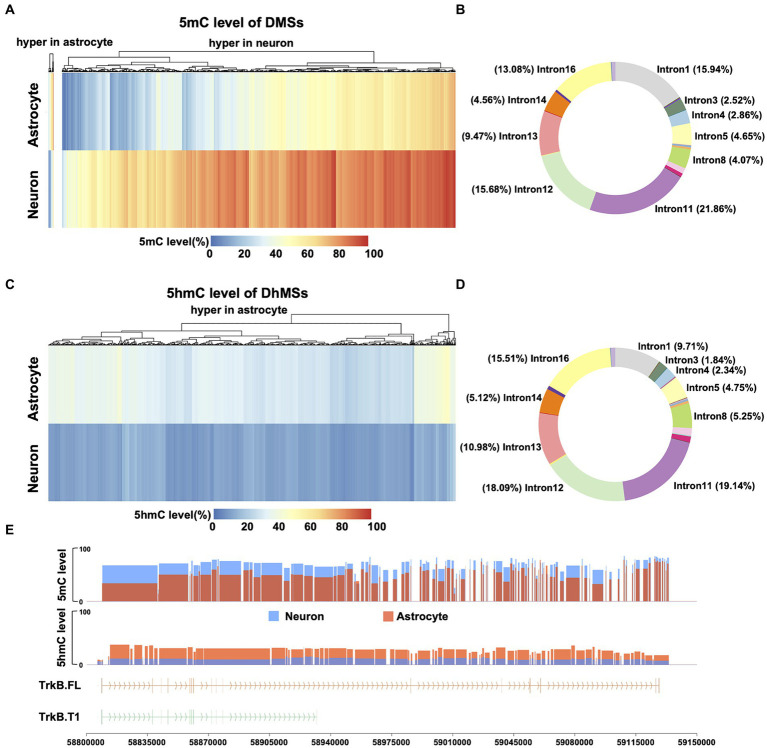
Comparison of 5mC and 5hmC of *Ntrk2* in astrocytes and neurons. **(A,C)** Heatmap of DMS **(A)** and DhMS **(C)** levels of *Ntrk2* in astrocytes and neurons. The colors represent the 5mC or 5hmC levels. **(B,D)** The distribution of DMSs **(B)** and DhMSs **(D)** on *Ntrk2*. **(E)** 5mC levels of DMRs and 5hmC levels of DhMRs identified between astrocytes and neurons. The vertical lines represent CDS regions in the gene structure cartoon and the arrows represent the direction of translation.

With the observed distinct differences in DNA 5mC and 5hmC modifications of *Ntrk2* between neurons and astrocytes, we next performed analysis across differentially methylated sites (DMSs) to determine where significant differences exist across the gene. Here, we defined a DMS as any single CpG site with a 5mC level difference greater than 10% (FDR <0.01) (Shi et al., 2021; Farlik et al., 2016). Comparing neurons to astrocytes, we identified 4,127 DMSs (Figure 3A) across the *Ntrk2* gene and promoter region. This analysis revealed 98.74% (4,074 of 4,127) of DMSs are hypermethylated in neurons relative to astrocytes. To determine the distribution of DMSs on gene *Ntrk2*, we annotated each DMS to its genomic regions: the promoter (1Kb upstream of the transcription start site), 5′UTR, 18 CDSs, 17 introns, and 3′UTR regions (Figure 3B). This quantitative analysis of methylation demonstrated there are no DMSs in the promoter region between astrocytes and neurons, perhaps unsurprising given the relatively high level of *Ntrk2* transcripts in both cell populations. Notably, we identified approximately 38% of all DMSs are annotated to intron 11 and 12 (107,504 bp, 32.8% of *Ntrk2*), which are the regions flanking the CDS12 (Figure 3B), the exon unique to the truncated transcript.

We next evaluated differentially hydroxymethylated sites (DhMSs), applying the same metrics as applied to identify DMSs any CpG site with a 5hmC level greater than 10% (FDR <0.01). Here, 100% (4,571) of DhMSs in astrocytes showed higher 5hmC level relative to neurons (Figure 3C). Annotation of each DhMS indicates that 37% of DhMSs are located in intron 11 and 12 (Figure 3D).

We next consolidated the DMSs/DhMSs to differentially (hydroxy) methylated regions (DMRs/DhMRs), which may reflect coordinated changes in 5mC/5hmC over a region that may serving to affect gene expression or regulatory elements. Here, these DMRs/DhMRs were defined as spanning over 50 bp, containing a minimum of 3 CpG sites, with ≥50% of CpG sites being DMSs/DhMSs (Figure 3E). Using this approach, we identified 106 DMRs and 80 DhMRs between astrocytes and neurons. Utilizing this approach, we observed 4.7%, (5/106) DMRs demonstrate hypermethylation in astrocytes relative to neurons, while 100% (80/80) of DhMRs are hypermethylated in astrocytes relative to neurons. Notably, all but one 5mC DMRs, located in intron 12, are hypermethylated in neurons relative to astrocytes. Together, these data indicate that differences in isoform expression between astrocytes and neurons may be derived from 5mC and 5hmC modifications circumscribed to the unique exon 12 of the *Ntrk2* gene.

## 4 Discussion

Decades of research have implicated BDNF/TrkB signaling in early brain development and neuronal maturation (Castren et al., 1992; Altar et al., 1997; An et al., 2008; Bramham and Messaoudi, 2005; Amaral and Pozzo-Miller, 2007; Wang et al., 1995). Further, adaptations to this signaling are observed in neurodevelopmental and neuropsychiatric conditions (Numakawa et al., 2018; Deng and Chen, 2024; Peng et al., 2009; Ferrer et al., 1999; Autry and Monteggia, 2012; Stansfield et al., 2012). Our recent work indicates that BDNF signaling through the astrocyte expressed truncated TrkB receptor is also critical for astrocyte morphological maturation (Holt et al., 2019), contributing to the impact of this signaling pathway in brain development. Despite its importance, very few studies have attempted to quantify differences in expression of TrkB across CNS cell types. Mining publicly available resources (Karlsson et al., 2021; Saunders et al., 2018; Human Protein Atlas, n.d.), we present data that across species TrkB expression is highest in astrocytes relative to other CNS cell populations. Additionally, the truncated isoform TrkB.T1 predominates in the human brain, corroborating our previous results demonstrating TrkB.T1 is more highly expressed in rodent cortex relative to TrkB.FL (Holt et al., 2019).

As it relates to isoform specific expression of TrkB, few studies have attempted to pinpoint the underlying molecular mechanisms. Early work demonstrated two alternative promoters in the TrkB gene in mouse brain. These promoters, designated P1 and P2, were subsequently demonstrated to play no role in distinct isoform expression, but instead generate mRNAs that are different in their 5’UTR region (Barettino et al., 1999). Interestingly, transcripts initiated for either the P1 or P2 promoter of *Ntrk2* generated both full length and truncated isoforms of the TrkB gene in cultured cortical neurons. Further, cAMP activation via CREB binding to a CRE element present in the P2 promoter, approximately 0.5 kb, upstream of the transcription start site of *Ntrk2*, triggered gene and protein expression of both TrkB isoforms (Deogracias et al., 2004). Although not yet evaluated, these results suggest CREB activation may serve to regulate TrkB.T1 expression in astrocytes. More recently, it was identified in human cortical tissue, collected post-mortem from suicide completers demonstrated lower levels of TrkB.T1 mRNA and protein. This finding correlated with altered methylation states of several CpG sites in the promoter region (Ernst et al., 2009) and 3′UTR region (Maussion et al., 2014), of the *NTRK2* gene. These findings indicate DNA methylation as a potential regulator of isoform specific TrkB expression, in line with a decade or more of work implicating DNA methylation as a regulator of exon inclusion or exclusion (Wen et al., 2014; Khare et al., 2012; Maunakea et al., 2013; Lev Maor et al., 2015; Shukla et al., 2011; Lopez Soto and Lipscombe, 2020).

Thus, here we assessed the methylation status of the full *Ntrk2* gene in enriched populations of cortical neurons and astrocytes; work that was facilitated by recent publication of Nanopore sequencing of mouse cortical astrocytes and neurons (Wei et al., 2024). Accessing these data we evaluated 5mC and 5hmC methylation across all 330 kb of *Ntrk2* in both cell types. This analysis revealed no differences in *Ntrk2* promoter methylation yet, thousands of differentially DMSs and DhMSs across the rest of the gene with enrichment of these epigenetic marks flanking CDS12, the TrkB.T1 specific isoform. Notably, 100% of DhMSs were hypermethylated in astrocytes relative to neurons, and correspondingly neurons demonstrated higher 5mC methylation. While correlative, these data suggest DNA methylation patterns may provide instruction for isoform specific TrkB expression across unique CNS cell types. Specifically, the high levels of 5mC and low levels of 5hmC in neurons may lead to the exclusion of CDS12, while the low levels of 5mC and high levels of 5hmC in astrocytes promote the inclusion of CDS12. This finding is consistent with previous publications indicating that DNA methylation levels are negatively correlated with TrkB.T1 expression levels (Ernst et al., 2009; Maussion et al., 2014). We observed that both intron 11 and intron 12, which flank the CDS12 region, exceed 5,000 bp in length. Although DMSs/DhMSs are not significantly overrepresented in these regions when normalized to genomic length, previous studies indicate that the prevalence and extent of alternative splicing are positively correlated with the mean intron size of the genome, suggesting that longer introns may contribute to alternative splicing events (Yang et al., 2021). Additionally, the RNA-binding gene Bruno-3 (*Bru-3*) in *Drosophila* has demonstrated that all exon-skipping events in this gene occur in exons flanked by introns of at least 800 nucleotides (Kandul and Noor, 2009). This observation is consistent with our findings that CDS12, is bordered by the long introns 11 and 12 and suggests that DNA methylation differences in these regions may facilitate alternative splicing. Together, data presented here provide additional insight regarding CNS TrkB expression across mice and humans and raise the possibility that DNA methylation may be a key driver of isoform specific expression across two unique cell populations.

While our study provides insights into the role of DNA methylation and hydroxymethylation in the regulation of alternative splicing and TrkB isoform expression, several limitations should be considered. First, our analysis is based on data from the P28 mouse cortex. Given that DNA methylation is dynamic throughout development, the methylation profile for *Ntrk2* could differ at other developmental stages. Second, our study utilized CO_2_ euthanasia, followed by cell isolation. While there is no definitive evidence indicating that CO_2_ euthanasia leads to rapid changes in the brain DNA methylome, it is important to acknowledge that DNA methylation patterns may be altered during the euthanasia and cell isolation processes, and that these changes may be cell type specific. Herein, our study corroborates previous work that DNA methylation may regulate TrkB isoform expression (Ernst et al., 2009, Maussion et al., 2014) and further supports the role of DNA methylation in the general regulation of isoform expression (Yearim et al., 2015; Ramanouskaya and Grinev, 2017; Shukla et al., 2011; Maunakea et al., 2013), yet our study does not establish causality between DNA methylation patterns and alternative splicing events of *Ntrk2*, necessitating further experimental validation.

## Data Availability

The original contributions presented in the study are included in the article/supplementary material, further inquiries can be directed to the corresponding author.
